# Clinical Robustness of FDG-PET/CT Quantitative Metrics Post-Harmonization in a Multicenter, Cross-Scanner Setting

**DOI:** 10.3390/tomography12070104

**Published:** 2026-07-13

**Authors:** Ayako Hino, Yoshinobu Ishiwata, Akira Kakiuchi, Tomohiro Numata, Hiroyuki Kamide, Hiroaki Kurihara, Zenjiro Sekikawa, Daisuke Utsunomiya

**Affiliations:** 1 Department of Diagnostic and Interventional Radiology, Kanagawa Cancer Center, 2-3-2 Nakao, Asahi-Ward, Yokohama 2418515, Japan; 2 Department of Diagnostic Radiology, Yokohama City University Medical Center, 4-57 Urafunecho, Minami-Ward, Yokohama 2320024, Japan; 3 Department of Nuclear Medicine, Yokohama City University Hospital, 3-9 Fukuura, Kanazawa-Ward, Yokohama 2360004, Japan; 4 Department of Diagnostic Radiology, Yokohama City University Hospital, 3-9 Fukuura, Kanazawa-Ward, Yokohama 2360004, Japan

**Keywords:** clinical PET imaging, biomarker robustness, multicenter, SUVmax, SUVpeak, MTV, inter-scanner harmonization

## Abstract

*Background/Objectives*: Differences among scanners and reconstruction methods may limit the comparability of quantitative metrics derived from fluorodeoxyglucose positron emission tomography (FDG PET)/computed tomography (CT). Although harmonization reduces inter-scanner variability in standardized uptake values (SUVs), its impact on the preservation of lesion-level ranking in real-world clinical datasets remains unclear. Here, we evaluated the robustness of PET quantitative metrics, particularly focusing on rank preservation after harmonization. *Methods*: Phantom and clinical data retrospectively acquired from three institutions using four PET/CT scanner types were analyzed. Harmonization parameters were derived from National Electrical Manufacturers Association IEC Body Phantom data, using the oldest scanner as the reference, and were directly applied to the clinical datasets. Clinical evaluation included head and neck malignant melanoma (HNMM; high FDG avidity) and adenoid cystic carcinoma (ACC; low FDG avidity). Rank preservation between pre- and post-harmonization values was assessed using Spearman’s rank correlation coefficient (ρ). *Results:* Phantom-based harmonization reduced inter-scanner differences and enabled consistent evaluation in clinical datasets (HNMM: 34 patients, 93 lesions; ACC: 18 patients, 38 lesions). SUVpeak demonstrated the highest rank preservation across tumor types and lesion sizes (ρ = 0.94–1.00). Metabolic tumor volume (MTV) showed a high rank correlation in HNMM with an absolute threshold (MTV2.5; ρ = 0.99), but robustness varied depending on threshold definition, tumor type, and lesion size. Tumor-to-liver ratios showed moderate rank preservation. *Conclusions:* Our results suggested that SUVpeak is the most robust preservation of lesion ranking across tumor types after harmonization, suggesting its suitability as a reliable imaging biomarker in multicenter studies. Meanwhile, careful standardization is needed when using MTV-based metrics for prognostic evaluation.

## Simple Summary

Differences among PET scanners and reconstruction methods can significantly affect quantitative PET metrics, including standardized uptake values. While harmonization reduces inter-scanner variability, its impact on lesion-level ranking, which is important for prognostic stratification, remains unclear. In this study, we applied phantom-based harmonization across multiple scanners and evaluated clinical data from head and neck cancers. We found that some PET metrics were susceptible to changes in lesion ranking after harmonization, whereas others remained stable. Notably, peak standardized uptake values consistently preserved lesion ranking across tumor types and lesion sizes, suggesting its potential as a robust imaging biomarker for multicenter studies.

## 1. Introduction

Quantitative positron emission tomography (PET) parameters play an important role in tumor prognostication and clinical decision-making. Metrics such as the maximum standardized uptake value (SUVmax), metabolic tumor volume (MTV), and total lesion glycolysis possess prognostic value across a wide range of malignancies and are therefore widely used as imaging biomarkers in clinical research and multicenter studies [1,2,3]. In recent years, advances in detector technology and image reconstruction techniques, especially the implementation of point spread function (PSF) modeling and adoption of silicon photomultiplier technology, have markedly improved the spatial resolution and lesion detectability of PET images [4,5,6]. However, quantitative values, including SUV, have been reported to show non-negligible inter-scanner variability depending on differences in image reconstruction methods [7]. When comparisons are performed without appropriate correction, such variability may compromise the consistency of clinical interpretation and prognostic assessment.

Harmonization has been widely adopted as an effective approach to ensure the comparability of quantitative PET metrics across different scanners [8,9,10,11]. In particular, phantom-based correction using physical recovery coefficients (RCs) represents an established method to minimize variability in RCs and SUVmax arising from differences in reconstruction settings and scanner performance [12,13]. Harmonization ranges proposed by organizations such as the European Association of Nuclear Medicine (EANM) and Japanese Society of Nuclear Medicine (JSNM) have been implemented as effective standards in multicenter studies [10,14]. Consequently, the inter-scanner correction of SUVmax is now a well-established technique and is widely applied in multicenter research [15,16]. For MTV, which is considered a quantitative biomarker of importance comparable to SUVmax, EANM Research Ltd. (EARL)-compliant harmonization reduces reconstruction-related variability and improves MTV consistency and delineation accuracy, whereas phantom studies have demonstrated that advanced reconstruction methods, such as Bayesian penalized likelihood reconstruction, further enhance MTV accuracy relative to true tumor volume [17,18].

However, for both SUVmax and MTV, existing studies have predominantly focused on harmonizing absolute quantitative agreement, whereas the systematic evaluation of whether the relative ranking of lesions is preserved pre- and post-harmonization remains limited. Here, “relative ranking” refers to the ordering of lesions within a disease cohort based on PET-derived parameters such as SUVs and MTV values, which is a key determinant in PET-based prognostic analyses. In diagnostic classification and prognostic models, the preservation of relative ranking of lesions or cases is critically important because substantial rank reversals or extreme outliers may cause model instability or even failure, particularly in multicenter analyses. In addition, the tumor-to-liver uptake ratio (T/L ratio) has been proposed as a relatively robust quantitative metric across different scanners and may serve as an alternative parameter in multicenter studies of rare cancers, wherein phantom-based harmonization is often difficult to implement [19,20]. Nevertheless, the behavior of the T/L ratio pre- and post-harmonization has not been sufficiently investigated.

In this context, the present study introduced ranking preservation as a primary evaluation axis and assessed fluorodeoxyglucose (FDG) PET quantitative metrics before and after phantom-based harmonization, with this concept defined as the main outcome. Specifically, we focused on two disease entities with similar anatomical locations but markedly different levels of FDG uptake: head and neck adenoid cystic carcinoma (ACC) and head and neck malignant melanoma (HNMM). Particular attention was paid to MTV, which was expected to exhibit behavior different from that of SUVmax owing to its strong dependence on segmentation methods, and to the T/L ratio, which has been regarded as a relatively robust quantitative metric. Furthermore, the study population was stratified into two groups from two perspectives: (1) differences in tumor FDG uptake intensity associated with biological differences between ACC and HNMM and (2) tumor physical size independent of tumor type. Using this stratification, we evaluated how these factors affected quantitative PET metrics and lesion-wise ranking post-harmonization. In addition, we investigated whether alternative quantitative metrics could serve as substitutes for harmonization in situations where phantom data are unavailable.

## 2. Materials and Methods

### 2.1. PET/CT Systems and Image Reconstruction Protocol

To simulate real-world multicenter studies of rare cancers requiring long-term accrual, we constructed a model incorporating both older and newer scanners. Phantom and clinical images were acquired using five PET systems across four PET/CT scanner types: Biograph Sensation 16 (hereafter Biograph 16; Siemens Medical Solutions, Erlangen, Germany), Biograph Horizon (Siemens Healthcare GmbH, Erlangen, Germany), Discovery IQ 4R (hereafter Discovery IQ; GE Healthcare, Milwaukee, WI, USA), and Discovery MI (GE Healthcare, Milwaukee, WI, USA). Discovery MI included two systems; phantom data were acquired using Discovery MI (A) shown in Table 1. Scanners were selected to encompass different generations and reconstruction algorithms commonly used in clinical practice, including ordered subset expectation maximization (OSEM) 2D, OSEM 3D with time of flight (TOF), and PSF-based methods. Images were reconstructed using standard clinical protocols (Table 1). Harmonization was performed using Biograph 16, which had the lowest image quality and quantitative performance among the evaluated systems, as the reference scanner. This approach prevented overestimation of quantitative values in newer systems and reduced inter-scanner variability, enabling more reproducible prognostic analyses.

### 2.2. Phantom-Based PET Harmonization

Phantom-based PET harmonization was performed using a NEMA NU2 image-quality phantom (ECT/IEC-BODY/P; Data Spectrum Corporation, Durham, NC, USA) containing six hot spheres with diameters ranging from 10 to 37 mm [21]. Details of phantom-adjustment procedures and data-analysis methods are provided in Appendix A.1.

### 2.3. SUV Measurements and Full Width at Half Maximum Selection

SUV measurements were performed pre- and post-harmonization to derive RCmax, RCmean, and noise-related metrics using the RAVAT software (ver1.02.00; Nihon Medi-Physics Co., Ltd., Tokyo, Japan). Details of procedures used to evaluate RCs and Root mean square error (RMSE), image quality and noise-related metrics, and full width at half maximum (FWHM) selections are provided in Appendix A.2. For image harmonization, we selected the smallest FWHM that fulfilled any of the reference standards (JSNM 3rd, JSNM 4th, EARL1, or EARL2) consistently across all acquisition durations [14,21,22]. MTV was calculated as MTV/Vtrue − 1 using an absolute SUV threshold of 2.5 (MTV2.5) and a relative threshold of 41% of SUVmax (MTV41%), both before and after SUV harmonization. Because no standardized harmonization criteria are available for MTV, volumetric measurements were not directly harmonized; instead, this analysis was designed to assess the indirect effects of SUV harmonization on MTV.

### 2.4. Visual Assessment Simulating Routine Clinical Interpretation

Visual assessment simulating routine clinical interpretation was performed using phantom images to identify the sphere sizes at which lesion conspicuity deteriorated. Pre- and post-harmonization images were anonymized and visually evaluated by four readers (two nuclear medicine physicians, one radiologist, and one first-year radiology resident) using a fixed SUV window (0–5) under standardized display conditions. Hot spheres (10–37 mm) were graded on a 4-point visibility scale, and inter-reader agreement was assessed using Fleiss’ κ. Based on this visual assessment, sphere sizes were classified according to deteriorated conspicuity and were subsequently used as surrogate indicators for small and large lesions in the quantitative analyses. Detailed evaluation procedures are provided in Appendix A.3.

### 2.5. Clinical Data

Data of patients with HNMM and ACC were retrospectively collected from three institutions. Radiology imaging databases and electronic medical records were searched to identify patients who had undergone PET/CT at diagnosis or before treatment initiation and follow-up PET/CT examinations using the target PET scanners between 2006 and 2025. Treatment regimens were not restricted because this study focused on the robustness of quantitative PET metrics rather than treatment response. Exclusion criteria were severe PET misregistration due to patient motion, incomplete image acquisition, and PET/CT examinations performed using scanners other than the four systems for which phantom data were available (Biograph 16, Biograph Horizon, Discovery IQ, and Discovery MI). Accordingly, the data of 34 patients with HNMM (14 males and 20 females; mean age 73.1 ± 11.2 years; primary sites: sinonasal in 33 cases and other in 1 case) and 18 patients with ACC (8 males and 10 females; mean age 61.2 ± 12.6 years; primary sites: salivary gland in 6 cases, nasal cavity in 5 cases, and other in 7 cases) were included.

### 2.6. Clinical Image Analysis

Primary tumors, lymph node metastases, and distant metastases were included in the image analysis, which was performed by two board-certified nuclear medicine physicians (A.H. and A.K.) using dedicated image analysis software (RAVAT). A spherical region of interest (ROI) was manually placed for each lesion, followed by semi-automatic segmentation of the MTV using an absolute SUV threshold of 2.5 and a relative threshold of 41% of SUVmax, based on the latest EANM guidelines and previous literature [10,23]. Subsequently, SUVmax, SUVpeak, and SUVmean were evaluated pre- and post-harmonization. SUVpeak was defined as the average SUV within a fixed 1-cm^3^ spherical volume positioned to yield the highest mean value [24]. For background assessment, a spherical ROI with a diameter of 3 cm was placed within the normal liver parenchyma, avoiding tumors and major vessels, and the T/L ratio was calculated.

### 2.7. Evaluation of Changes in Quantitative PET Parameters Post-Harmonization

The preservation of paired lesion-wise ranking (i.e., ordering of lesions within a cohort based on quantitative PET values) pre- and post-harmonization was evaluated for each parameter. Rank preservation was assessed using Spearman’s rank correlation coefficient and the absolute value of rank change. Separate analyses were performed by tumor type (ACC and HNMM) and by lesion size to evaluate ranking preservation. Pearson’s correlation coefficient was used only to evaluate linear associations between absolute values, whereas ranking preservation was assessed using Spearman’s correlation and absolute rank changes.

### 2.8. Statistical Analyses

Statistical analyses were performed using Microsoft Excel, SPSS (version 30.0.0.0 [172]; IBM Corp., Armonk, NY, USA), JMP Student Edition (version 19; JMP Statistical Discovery LLC, Cary, NC, USA), and R (version 4.x; Foundation for Statistical Computing, Vienna, Austria). κ values, 95% confidence intervals, and κ differences were estimated with the irr package using nonparametric bootstrap resampling (10,000 iterations) at the sphere level [25].

## 3. Results

### 3.1. Phantom Experiments

#### 3.1.1. RC Evaluation and Determination of Harmonization Targets

**Figure A1:**
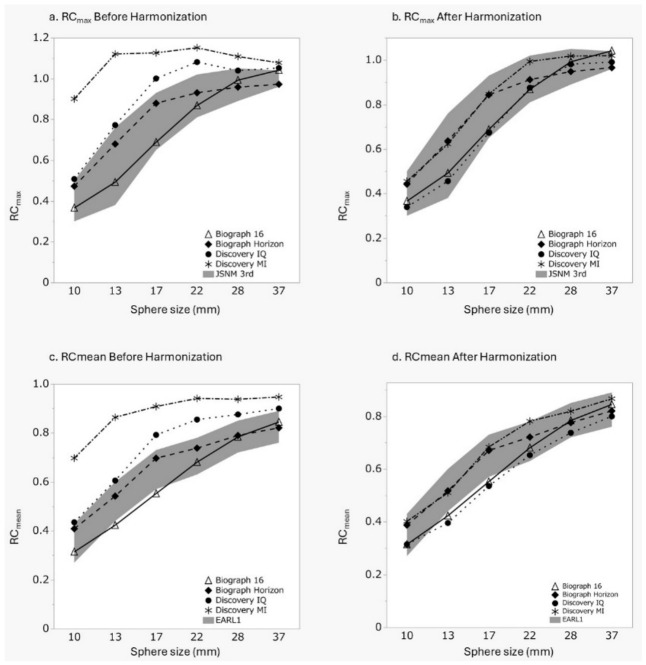
Recovery coefficients (RCs) before and after harmonization (30-min acquisition). (**a**) RCmax values from all systems plotted with the JSNM 3rd reference range. Biograph 16 and Biograph Horizon met the JSNM 3rd criteria, whereas Discovery IQ and Discovery MI did not. (**b**) After harmonization, RCmax values of all systems converged toward those of Biograph 16 (lowest RCmax) and fell within the JSNM 3rd reference range. (**c**) RCmean values plotted with the EARL1 reference range. For Biograph 16, the 13-mm and 17-mm spheres were below the lower EARL1 limit, whereas Discovery IQ and Discovery MI exceeded the upper limit across all sphere sizes. Biograph Horizon met the EARL1 criteria for all spheres. (**d**) After harmonization, RCmean values shifted toward those of Biograph 16, improving inter-scanner consistency.

Because Biograph 16 met only the JSNM 3rd edition RCmax criteria, this standard was selected as the harmonization target. FWHM values satisfying the JSNM3rd reference range for each PET/CT system were 0 mm for Biograph 16, 4 mm for Biograph Horizon, 9 mm for Discovery IQ, and 8 mm for Discovery MI. Inter-scanner differences in RCmax and RCmean observed pre-harmonization were reduced post-harmonization. Detailed comparisons of RCmax and RCmean pre- and post-harmonization are shown in Figure A1 and Appendix B.1, where the results of the FWHM fitting are also presented (Table A1).

#### 3.1.2. Evaluation of Root Mean Square Error, MTV Accuracy, and Image Quality and Noise

**Figure 1:**
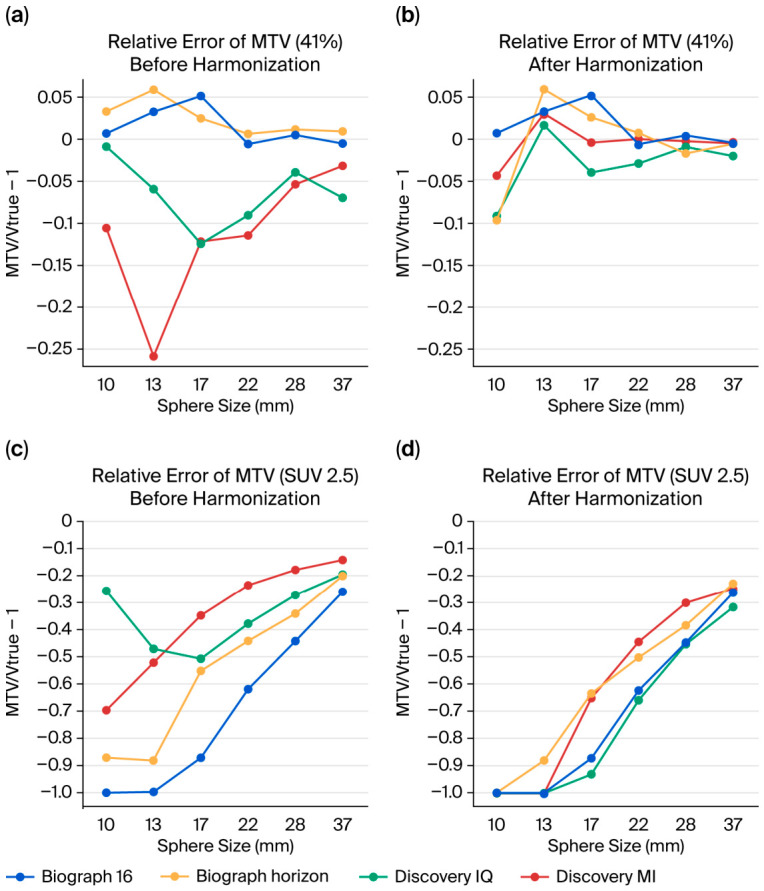
Relative error of MTV, expressed as MTV/Vtrue − 1, for each sphere size pre- and post-harmonization. (**a**) 41% SUVmax threshold pre-harmonization; Discovery IQ and Discovery MI showed negative errors for small spheres. (**b**) 41% SUVmax threshold post-harmonization. Inter-scanner differences were reduced. (**c**) Absolute SUV 2.5 threshold pre-harmonization. (**d**) Absolute SUV 2.5 threshold post-harmonization. MTV values converged toward those of Biograph 16. For the 10-mm sphere, SUVmax fell below 2.5 for all scanners, resulting in MTV = 0.

**Figure A2:**
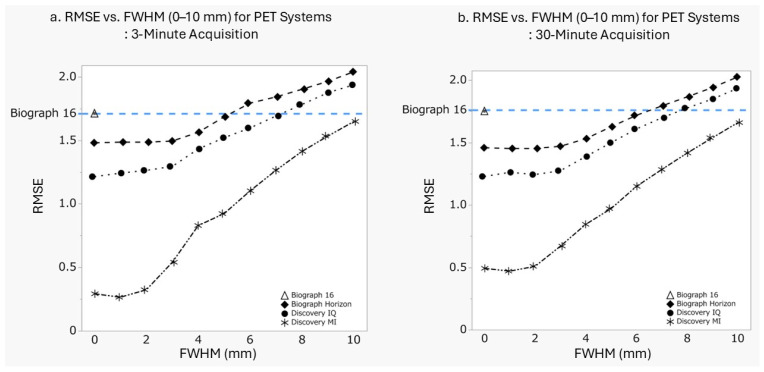
RMSE vs. FWHM (0–10 mm) for PET Systems. The light blue horizontal line extending from the triangle represents the RMSE of the Biograph 16 scanner. (**a**) Three-minute acquisition. At FWHM = 0, RMSE was highest for Biograph 16, followed by Biograph Horizon, Discovery IQ, and Discovery MI. RMSE values comparable to Biograph 16 were observed at FWHM = 5–6 mm for Biograph Horizon and 7–8 mm for Discovery IQ, whereas Discovery MI remained lower than Biograph 16 across all FWHM values. (**b**) Thirty-minute acquisition. Comparable RMSE values to Biograph 16 were observed at FWHM = 6–7 mm for Biograph Horizon and 7–8 mm for Discovery IQ. Discovery MI remained lower than Biograph 16 throughout the full FWHM range.

RMSE increased with increasing FWHM post-harmonization across all PET/CT systems (Figure A2). Inter-scanner differences in MTV measurements were reduced post-harmonization (Figure 1).

MTV41% showed a tendency toward overestimation on older scanners and underestimation on newer scanners, likely reflecting scanner-dependent differences in recovery coefficients affecting relative thresholding. Meanwhile, MTV2.5 for small spheres could not be evaluated on Biograph 16, and reduced SUVs post-harmonization further limited MTV2.5 assessment on the other scanners. Despite these effects, background-noise metrics post-harmonization satisfied the JSNM-recommended reference ranges for all scanners and acquisition durations. Detailed phantom results (Appendix B.1), including RMSE (Appendix B.2), the results of the FWHM fitting are also presented (Appendix B.3, Table A1), and noise metrics pre- and post-harmonization (Appendix B.4, Table A2), are provided in Appendix B.

#### 3.1.3. Visual Analysis

Among all readers (*n* = 4), the overall Fleiss’ κ was 0.58 (0.59 pre- and 0.54 post-harmonization), indicating moderate agreement. For larger spheres (≥22 mm), agreement was substantial to moderate (κ = 0.61 overall, 0.68 before, and 0.55 post-harmonization), whereas for smaller spheres, agreement was lower and decreased post-harmonization (κ = 0.36 overall, 0.44 before, and 0.13 after).

To assess the effect of reader experience, a secondary analysis excluding the trainee (*n* = 3) was performed. Overall agreement increased to almost perfect (κ = 0.86 overall, 0.90 pre- and 0.82 post-harmonization). For larger spheres (≥22 mm), κ values remained high (0.97 overall, 1.0 pre-, and 0.94 post-harmonization), whereas for smaller spheres, agreement ranged from substantial to almost perfect but again decreased post-harmonization (0.76 overall, 0.81 pre-, and 0.66 post-harmonization). Based on these findings, a lesion diameter of 22 mm was defined as the threshold between small and large lesions.

### 3.2. Clinical Data Analysis

#### 3.2.1. Clinical Parameters

In total, 93 lesions from HNMM cases and 38 lesions from ACC cases were included. Imaging sites, lesion-size distribution, and scanner allocation are summarized in Table 2. For one patient with ACC, both baseline and follow-up scans were included; therefore, the number of primary tumor site counts exceeded the total number of patients by one.

Quantitative FDG PET parameters pre- and post-harmonization are shown in Table 3; data from Biograph 16 were excluded from pre- and post-harmonization comparisons because images were reconstructed with an FWHM of 0 mm. Because FDG uptake was generally higher in HNMM than in ACC, HNMM was classified as an FDG-avid tumor and ACC as a low-FDG uptake tumor.

#### 3.2.2. Changes in PET Parameters Pre- and Post-Harmonization in FDG-Avid HNMM

**Figure 2:**
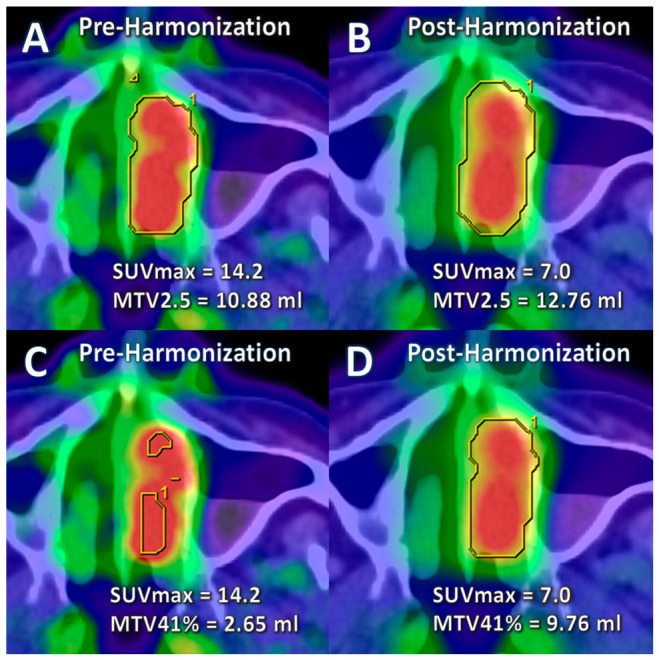
MTV segmentation using absolute (MTV2.5) and relative (MTV41%) thresholds in a representative case of HNMM, before and after harmonization. (**A**) MTV2.5 pre-harmonization; (**B**) MTV2.5 post-harmonization; (**C**) MTV41% pre-harmonization; and (**D**) MTV41% post-harmonization. MTV increased after harmonization with both absolute (2.5) and relative (41%) segmentation methods. The number “1” indicates the VOI label automatically displayed by the software and cannot be removed.

In the HNMM group, rank changes in PET parameters pre- and post-harmonization were evaluated separately for single-scanner (Discovery MI) and multi-scanner (Discovery MI and Biograph 16) datasets. All parameters showed significant differences between pre- and post-harmonization (paired *t*-test). Most PET parameters decreased post-harmonization, except for MTV2.5 and MTV41%. Representative examples of MTV segmentation using MTV2.5 and MTV41% pre- and post-harmonization are shown in Figure 2.

Rank changes in PET parameters in the single scanner analysis using Discovery MI (57 lesions; values shown as median [mean]) are summarized in Table 4.

**Figure 3:**
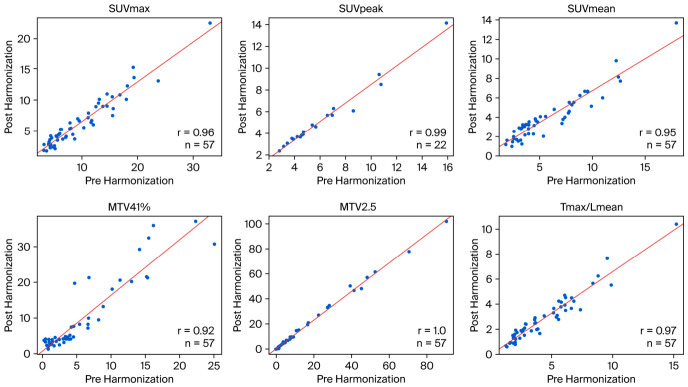
Pearson correlation scatter plots of PET parameters before and after harmonization in patients with HNMM using a single scanner (Discovery MI). The red line represents the fitted linear regression line. Scatter plots and Pearson correlation coefficients for pre- and post-harmonization semiquantitative parameters (SUVmax, SUVpeak, SUVmean, MTV41%, MTV2.5, Tmax/Lmean) on Discovery MI. The x- and y-axes represent pre- and post-harmonization values, respectively. Strong linear correlations were observed for SUVpeak and MTV2.5, while MTV41% showed greater variability with outliers. SUVmax, SUVmean, and Tmax/Lmean also showed good correlations.

SUVpeak showed the smallest rank change (1 [0.73], 22 lesions), followed by MTV2.5 (1 [1.53]), tumor maximum-to-liver mean uptake ratio (Tmax/Lmean) (3 [4.07]), SUVmax (4 [4.42]), and tumor-to-liver maximum uptake ratio (Tmax/Lmax) (4 [4.49]). In contrast, MTV41% (4 [5.61]) and SUVmean (4 [6.21]) exhibited relatively greatest variability. Spearman’s analysis showed near perfect rank preservation for SUVpeak and MTV2.5 (ρ = 0.99), whereas MTV41% was the only parameter with a coefficient below 0.9 (ρ = 0.88). Pearson’s correlation analysis showed near perfect linear correlations for SUVpeak and MTV2.5, with MTV41% again showing weaker correlation (Figure 3).

Dual-scanner analysis combining Discovery MI and Biograph 16 (93 lesions; values shown as median [mean]) is also summarized in Table 4. SUVmax exhibited pronounced rank variability (10 [9.48]), which was greater than that observed in the single-scanner analysis. SUVpeak (1 [1.53], 38 lesions) showed the smallest rank change, followed by MTV2.5 (1 [2.25]). Although SUVpeak has limited measurability in small lesions, post-harmonization Spearman’s analysis demonstrated near-perfect rank preservation (ρ = 0.99). In contrast, MTV41% was the only parameter with a correlation coefficient below 0.9 (ρ = 0.89). The overall trends observed in the multi-scanner analysis were largely comparable to those seen in the Discovery MI–only analysis for the other parameters).

#### 3.2.3. Changes in PET Parameters Pre- and Post-Harmonization in Low FDG Uptake ACC (Exploratory Analysis)

As an exploratory analysis, PET parameters were evaluated in 18 patients with ACC, comprising 38 lesions imaged using 3 PET/CT systems (Biograph Horizon, Discovery IQ, and Discovery MI). Actual measured values are presented in Table 3. All evaluated parameters except MTV2.5 showed significant differences between pre- and post-harmonization. Notably, MTV2.5 became zero after harmonization in 14 cases, indicating that the lesions were not detectable using this threshold, and no significant difference in MTV2.5 were noted after harmonization (paired *t*-test, *p* = 0.84). In contrast to that in HNMM, only MTV41% increased post-harmonization, whereas all other parameters decreased.

Rank changes in PET parameters pre- and post-harmonization in ACC are summarized in Table 4. Although SUVpeak could be assessed in only a limited number of lesions (9 lesions), rank changes were completely preserved (0 [0]), with Spearman’s rank correlation coefficient of 1.0, indicating perfect rank consistency among evaluable cases. In contrast, MTV2.5 (1 [5.05]) and MTV41% (3 [4.37]) showed greater rank variability than that in HNMM, despite the smaller number of lesions. Spearman’s rank correlation analysis demonstrated very high correlations for SUV-based parameters, with coefficients of 1.0 for SUVpeak and ≥0.95 for SUVmax, SUVmean, and T/L ratios. In contrast, rank correlations for MTV-based parameters were substantially lower, with both MTV41% and MTV2.5 showing coefficients below 0.9. Consistent with these findings, Pearson’s correlation analysis demonstrated strong linear correlations (r > 0.9) for all PET parameters except MTV41%. Notably, although rank preservation for MTV2.5 was reduced, its Pearson correlation coefficient remained very high (r = 0.99), indicating a discrepancy between rank stability and linear correlation.

#### 3.2.4. Changes in PET Parameters Pre- and Post-Harmonization Between Large (≥22 mm) and Small Lesions (<22 mm)

Regardless of tumor type, lesions were classified as small (<22 mm) or large (≥22 mm) based on phantom studies demonstrating reduced conspicuity for spheres < 22 mm. Lesions acquired with the Biograph 16 scanner (FWHM = 0 mm), which showed no numerical changes post-harmonization, were excluded from the quantitative analysis, yielding 95 lesions (large, *n* = 37; small, *n* = 58). Pre-harmonization, all quantitative PET parameters were higher in large lesions than in small lesions. Post-harmonization, MTV41% increased in both groups, and MTV2.5 increased only in large lesions; all other parameters decreased (Table 5).

Rank changes between pre- and post-harmonization values were assessed using the full dataset without scanner-based exclusion (131 lesions: large, *n* = 56; small, *n* = 75) (Table 6). In both groups, SUVpeak and MTV2.5 showed minimal rank changes.

In small lesions, Spearman’s rank correlation coefficients (ρ) were highest for SUVpeak (ρ = 0.94) and MTV2.5 (ρ = 0.93), followed by Tmax/Lmax (ρ = 0.90), whereas all other parameters were <0.9. Notably, MTV41% showed markedly reduced rank stability (ρ = 0.61), consistent with lower Pearson correlation coefficients (r).

In large lesions, rank preservation was generally stronger. Rank changes were smallest for SUVpeak (ρ = 0.98) and MTV2.5 (ρ = 0.99). For MTV41%, both rank changes and correlation coefficients (Spearman’s ρ = 0.92; Pearson’s r = 0.92) were improved compared with those for small lesions, indicating greater rank stability with increasing lesion size.

## 4. Discussion

### 4.1. Phantom-Based Harmonization

Phantom experiments demonstrated that RC values were well aligned within the JSNM reference range, accompanied by reduced inter-scanner variability in MTV metrics. While MTV41% improved inter-scanner consistency, MTV2.5 occasionally resulted in nonmeasurable MTVs in small spheres. In contrast, the reader study showed a decline in accuracy for spheres ≤ 22 mm, despite the use of a uniform phantom. These findings suggest that, particularly in small or low-uptake lesions, both visual assessment and quantitative measurements may be subject to observer dependency. This limitation may be especially relevant in anatomically complex regions such as the head and neck, as well as in lymph node evaluations where false positives are common.

### 4.2. Clinical Data Evaluations

#### 4.2.1. Changes in PET Parameters Pre- and Post-Harmonization in FDG-Avid HNMM

Pre- and post-harmonization, SUVmax showed substantial rank variability even in the single-scanner comparison (57 lesions), with a median rank change of 4 (mean 4.42). In the multi-scanner cohort (93 lesions), the median and mean rank changes were 10 and 9.48, respectively. Overall, rank changes occurred in approximately 7–9.3% of lesions. These shifts may affect stratification, particularly in small cohorts. One possible explanation is that high-resolution reconstruction techniques, such as PSF and TOF implemented in Discovery MI, increase sensitivity to outliers in small lesions, thereby amplifying rank fluctuations [26].

These results suggest that when prognostic analyses based on SUVmax are performed using PET images acquired with different scanner generations and reconstruction methods, without harmonization, cutoff values are prone to shift, which may in turn affect the optimal thresholds used for clinical decision-making. In other words, harmonization is essential for ensuring reliable prognostic evaluation. Houdu et al. reported that differences in reconstruction conditions can lead to changes in the optimal SUVmax cutoff even within the same patients, potentially resulting in a loss of prognostic performance [27]. These findings are consistent with the results of the present study, indicating that clinical decisions based on SUV measurements may be influenced by scanner-dependent variability and supporting the necessity of harmonization. In contrast, MTV2.5 and SUVpeak showed minimal rank changes, indicating higher stability in FDG-avid tumors, although SUVpeak has limitations in small lesions. MTV41%, defined by a relative threshold, showed greater rank variability, likely reflecting its sensitivity to inter-scanner differences in SUV recovery. Previous studies have also reported that MTV retains prognostic value across thresholding methods, with fixed thresholds providing more consistent performance than relative thresholds; these findings are supported by the present results even post-harmonization [28]. Tmax/Lmean and Tmax/Lmax also showed relatively high rank correlations; however, their stability was inferior to that of MTV2.5 and SUVpeak.

#### 4.2.2. Changes in PET Parameters Pre- and Post-Harmonization in Low FDG Uptake ACC

ACC is characterized by lower FDG uptake compared to HNMM, and distinct differences in rank changes post-harmonization were observed. Overall, SUVpeak maintained high rank stability even in low-FDG-avid tumors, whereas MTV2.5 and MTV41% showed greater variability, indicating the need for the cautious interpretation of MTV-based metrics [28].

MTV2.5 showed reduced rank stability in ACC than in HNMM, mainly owing to lesions with low SUVmax in which MTV2.5 became zero post-harmonization. Despite this observation, Pearson correlation remained high, suggesting a discrepancy between rank stability and linear correlation that likely reflected the small absolute variation in uptake in low-FDG lesions. MTV41% showed a similar tendency with relatively large rank variability. In contrast, SUVpeak demonstrated perfect rank agreement, although evaluable lesions were limited, supporting its robustness even under low-contrast conditions. SUVmax-based parameters (Tmax/Lmean, Tmax/Lmax, SUVmax, SUVmean) also showed high rank correlations, with trends comparable to those in HNMM.

#### 4.2.3. Changes in PET Parameters Pre- and Post-Harmonization in Large (≥22 mm) and Small lesions (<22 mm)

Using 22 mm—identified in our phantom experiments as the threshold at which lesion conspicuity declines—as the cutoff for lesion-size classification, quantitative PET parameters were generally higher in large lesions than in small lesions, consistent with reduced lesion detectability. Among SUV-based parameters, SUVpeak showed the smallest variability and the highest rank preservation in both large and small lesions, indicating that it is the most stable metric. In contrast, for MTV metrics, the absolute-threshold parameter (MTV2.5) demonstrated high rank preservation in both groups, whereas the relative-threshold parameter (MTV41%) showed reduced rank stability. This instability was particularly pronounced in small lesions, suggesting that relative-threshold methods are more susceptible to intratumoral heterogeneity and image noise. These findings indicate that, when FDG uptake in relatively small primary tumors or lymph node lesions is used for prognostic assessment, the reliability of cutoffs based on MTV41% may be compromised. SUVmax, SUVmean, Tmax/Lmean, and Tmax/Lmax showed similar degrees of rank variability in both large and small lesions and did not demonstrate the same level of stability as SUVpeak or MTV2.5. This may be attributable to effects of RC correction, particularly in metrics based on maximum values, which are more susceptible to correction-related fluctuations.

### 4.3. Clinical Implications of Harmonization for HNMM and ACC

MTV2.5 increased in HNMM but decreased in ACC, indicating different post-harmonization behavior depending on FDG avidity. In HNMM, high uptake results in many voxels being near the threshold, and smoothing with a large FWHM Gaussian kernel increases peripheral SUV (spill-in), leading to more voxels exceeding the threshold. In contrast, in ACC, lower uptake, reduced contrast, and partial-volume effects after harmonization cause more voxels to fall below 2.5, resulting in decreased MTV2.5. Similarly, MTV41% may be overestimated in high-uptake lesions and underestimated in low-uptake lesions after harmonization, reflecting its dependence on relative thresholding.

Overall, the impact of harmonization appears greater in tumors with low FDG uptake and in small lesions. Clinically, this is particularly relevant in head and neck cancers, where a tumor size of 2 cm corresponds to a transition from T1 to T2. As lesion size is closely linked to staging and FDG uptake, harmonization-related variability may confound interpretation if not properly addressed. Therefore, careful case selection and statistical adjustment for covariates such as tumor stage and lesion size are needed in prognostic and multicenter analyses. When cutoffs (e.g., ROC or median) are used for prognostic analyses, PET metrics may be sensitive to harmonization, particularly near thresholds; this may cause classification instability and rank changes. Although no cutoff was defined in this study, this remains an important consideration in PET-based prognostic assessment.

Both MTV2.5 and SUVpeak showed detectability limitations in some lesions but demonstrated high robustness when measurable. These findings suggest that they may serve as practical alternative metrics, particularly in larger, highly FDG-avid tumors, when phantom-based harmonization is not feasible. Most evidence supporting SUVpeak has been derived from single-center or standardized imaging settings [29,30], whereas our findings highlight its reproducibility across heterogeneous multicenter conditions, supporting its use in rare disease studies.

### 4.4. Limitations

This study has some limitations. First, the Biograph 16 used is an older-generation PET system, and phantom experiments were conducted based on earlier JSNM protocols; therefore, the study does not fully comply with the latest JSNM and EARL guidelines. In addition, harmonization was performed using an older system as the reference, and the behavior of quantitative parameters based on contemporary standards (JSNM 4th, EARL2) was not evaluated. Moreover, as the oldest system, the Biograph 16 is no longer available, making additional phantom experiments infeasible. However, retrospective real-world studies of rare cancers require long-term integration of multicenter PET images acquired with different scanners. The findings of this study provide a potential approach to address challenges encountered in such real-world, multicenter retrospective studies. Second, to illustrate high- and low-uptake tumors, HNMM and ACC were selected as representative entities; however, both are rare diseases, resulting in a limited sample size. Clinical comparison for HNMM was restricted to two PET systems, while although multi-scanner harmonization was feasible for ACC, the number of cases per scanner was small. Moreover, despite its robustness, SUVpeak is difficult to measure in small lesions, potentially limiting evaluable cases and highlighting a trade-off between robustness and clinical applicability. Finally, absolute threshold-based metrics such as MTV2.5 are influenced by tumor FDG uptake and may be less reliable in low-uptake tumors or post-treatment lesions.

## 5. Conclusions

In this study, phantom-based harmonization was performed using an older-generation PET system as the reference, and the robustness of quantitative PET parameters pre- and post-harmonization was evaluated in rare cancers, including ACC and HNMM. SUVpeak showed the highest robustness across lesion sizes and FDG uptake levels, despite limited applicability in small lesions. MTV2.5 also demonstrated relatively high stability, except in low-FDG-avid tumors. In contrast, SUVmax showed substantial variability and was strongly affected by RC correction. Tmax/Lmax and Tmax/Lmean preserved lesion ranking to some extent but were less robust than SUVpeak and MTV2.5. Overall, these findings highlight the importance of appropriate PET parameter selection in harmonized datasets, particularly in prognostic and multicenter analyses.

## Figures and Tables

**Table 1:** Imaging and reconstruction parameters of PET scanners.

System	Biograph 16	Biograph Horizon	Discovery IQ	Discovery MI (A) *	Discovery MI (B)
Vendor	Siemens Healthineers	Siemens Healthineers	GE Healthcare	GE Healthcare	GE Healthcare
Detector type	LSO	LSO	BGO	SiPM	SiPM
Reconstruction method	OSEM 2D	OSEM3D + TOF	Q.Clear (PSF)	Q.Clear (TOF + PSF)	Q.Clear (TOF + PSF)
Reconstruction parameter	it. 2, sub. 8	it. 4, sub. 10	β350	β400	β400
Matrix Size	168 × 168	180 × 180	192 × 192	256 × 256	256 × 256
Pixel size (transaxial, mm)	4.1 × 4.1	4.1 × 4.1	3.6 × 3.6	2.0 × 2.0	2.8 × 2.8
min/bed position	2	1.75	2	2	2
Slice Thickness (mm)	5	5	3.3	2.8	3
Smoothing	Gaussian 5 mm	Gaussian 5 mm	N/A	N/A	N/A

Abbreviations: Lutetium oxyorthosilicate (LSO); Bismuth germanate (BGO); Ordered subset expectation maximization (OSEM); Time of flight (TOF); Q.Clear: GE Healthcare Bayesian penalized likelihood image reconstruction algorithm.; not applicable (N/A) * Phantom data for both Discovery MI systems were derived from only one of the two scanners (A), which served as the representative system.

**Table 2:** Lesion characteristics from HNMM and ACC.

Tumor Characteristics ^1^		HNMM (*n* = 93)	ACC (*n* = 38)
Primary		34	19
Metastatic	Lung	7	16
	Bone	11	0
	Liver	23	0
	Lymph nodes	18	3
Tumor size (mm)	Mean (SD)	27.7 (19.0)	22.5 (17.8)
	Median	20	14
Small lesion ^2^	<22 mm	49	26
Large lesion ^2^	≥22 mm	44	12
Scanner used for lesion acquisition, *n*			
	Biograph 16	36	N/A
	Biograph Horizon	N/A	5
	Discovery IQ	N/A	20
	Discovery MI	57	13

^1^ Tumor characteristics and scanner usage are reported per lesion; ^2^ A cutoff of 22 mm was used based on phantom experiments showing a change in lesion visibility; not applicable (N/A).

**Table 3:** Lesion-based PET parameters of HNMM and ACC pre- and post-harmonization.

	HNMM (*n* = 57) ^1^			ACC (*n* = 38)		
	Pre- Harmonization	Post- Harmonization	Δ	Pre- Harmonization	Post- Harmonization	Δ
SUVmax	9.7 (5.9)	6.3 (4.0)	−3.5 (2.3)	4.6 (3.2)	3.1 (2.4)	−1.54 (1.30)
SUVpeak ^2,3^	6.0 (3.1) ^3^	5.8 (3.6) ^3^	−0.9 (0.6)	5.2 (3.0) ^3^	3.7 (2.3) ^3^	−0.45 (0.41)
SUVmean ^2^	5.8 (2.2)	4.0 (2.3)	−1.8 (1.4)	2.7 (1.8)	1.9 (1.4)	−0.81 (0.72)
MTV41%	5.31 (5.62)	9.0 (9.46)	3.70 (4.75)	6.28 (8.64)	9.49 (10.49)	3.18 (4.73)
MTV2.5	11.69 (18.73)	13.21 (21.41)	1.53 (2.92)	6.73 (12.8)	6.51 (13.21)	−0.39 (2.58)
Tmax/Lmean	4.27 (2.64)	2.77 (1.81)	−1.50 (1.01)	1.90 (1.52)	1.47 (1.12)	−0.63 (0.56)
Tmax/Lmax	3.28 (2.07)	2.43 (1.6)	−0.84 (0.67)	1.49 (1.23)	1.27 (0.99)	−0.15 (0.37)

Unless otherwise indicated, all values are expressed as mean (SD). Δ indicates the difference between pre- and post-harmonization values. ^1^ data from Biograph 16 were excluded from pre- and post-harmonization comparisons because images were reconstructed with an FWHM of 0 mm; ^2^ SUVpeak and SUVmean were calculated using an MTV threshold of 41%; ^3^ Missing SUVpeak values: HNMM, 41 pre- and 33 post-harmonization; ACC, 29 pre- and 17 post-harmonization.

**Table 4:** Rank changes in PET parameters pre- and post-harmonization in HNMM and ACC.

	HNMM								ACC			
	Single Scanner ^1^				Multi-Scanners ^2^				Multi-Scanners ^3^			
PET Parameters	*n*	|ΔRank| ^4^	ρ	r	*n*	|ΔRank| ^4^	ρ	r	*n*	|ΔRank| ^4^	ρ	r
SUVmax	57	4 [4.42]	0.94	0.96	93	10 [9.48]	0.91	0.92	38	1 [1.95]	0.95	0.93
SUVpeak	22	1 [0.73]	0.99	0.99	38	1 [1.53]	0.99	0.99	9	0 [0.00]	1	1
SUVmean	57	4 [6.21]	0.95	0.95	93	9 [8.90]	0.91	0.93	38	1 [2.11]	0.95	0.93
MTV41%	57	4 [5.61]	0.88	0.92	93	6 [9.40]	0.89	0.95	38	3 [4.37]	0.84	0.89
MTV2.5	57	1 [1.53]	0.99	1	93	1 [2.25]	0.99	1	38	1 [5.05]	0.82	0.99
Tmax/Lmean	57	3 [4.07]	0.95	0.97	93	8 [8.60]	0.93	0.95	38	1 [1.84]	0.97	0.96
Tmax/Lmax	57	4 [4.49]	0.94	0.96	93	7 [7.18]	0.94	0.93	38	1 [1.84]	0.96	0.93

Values are presented as Spearman’s rank correlation coefficients (ρ) and Pearson’s correlation coefficients (r). All *p*-values were <0.0001. ^1^ Single-scanner: Discovery MI; ^2^ multi-scanner (two systems): Discovery MI + Biograph 16; ^3^ multi-scanner (three systems): Discovery MI + Biograph Horizon + Discovery IQ; ^4^ Rank change (|ΔRank|) was defined as the absolute difference in lesion-wise rank between pre- and post-harmonization and is summarized as median [mean].

**Table 5:** Comparison of PET parameters between large and small lesions pre- and post-harmonization.

Total (*n* = 95)	Small Lesions (<22 mm) (*n* = 58)			Large Lesions (≥22 mm) (*n* = 37)		
	Pre- Harmonization	Post- Harmonization	Δ	Pre- Harmonization	Post- Harmonization	Δ
SUVmax	5.0 (3.0)	3.0 (1.8)	−2.0 (1.5)	11.9 (6.1)	8.1 (4.0)	−3.8 (2.6)
SUVpeak *	4.1 (1.1) **	2.8 (1.2) **	−0.5 (0.3)	7.2 (3.4) **	7.1 (3.4) **	−1.0 (0.7)
SUVmean *	3.1 (1.9)	2.0 (1.1)	−1.1 (1.0)	6.9 (3.5)	4.9 (2.4)	−2.0 (1.4)
MTV41% (mL)	2.50 (1.96)	4.18 (2.19)	1.68 (1.48)	10.71 (8.83)	17.08 (11.85)	6.4 (6.44)
MTV2.5 (mL)	2.43 (2.07)	1.68 (1.02)	−0.01 (0.48)	23.53 (23.50)	24.90 (24.29)	3.49 (3.53)
Tmax/Lmean	2.20 (1.27)	1.36 (0.78)	−0.84 (0.61)	5.28 (2.72)	3.64 (1.80)	−1.64 (1.18)
Tmax/Lmax	1.67 (1.02)	1.18 (0.69)	−0.49 (0.45)	4.08 (2.11)	3.19 (1.60)	−0.88 (0.75)

Biograph 16 data with FWHM = 0 were excluded from the analysis. Unless otherwise indicated, all values are expressed as mean (SD). Δ indicates the difference between pre- and post-harmonization values. * SUVpeak and SUVmean were calculated using an MTV threshold of 41%; ** Missing SUVpeak values: small lesions, 44 pre-and 28 post-harmonization; large lesions (>22 mm), 20 pre-and 2 post-harmonization.

**Table 6:** Rank change pre- and post-harmonization in large and small lesions.

Total (*n* = 131)	Small Lesions (<22 mm) (*n* = 75)				Large Lesions (≥22 mm) (*n* = 56)			
PET Parameters	*n*	|ΔRank| *	ρ	r	*n*	|ΔRank| *	ρ	r
SUVmax	75	10 [12.09]	0.85	0.87	93	8 [9.38]	0.89	0.9
SUVpeak	58	2 [2.18]	0.94	0.98	20	1 [1.63]	0.98	0.99
SUVmean	75	11 [12.51]	0.85	0.85	93	9 [9.57]	0.9	0.93
MTV41%	75	13 [17.84]	0.61	0.85	93	6 [9.64]	0.92	0.92
MTV2.5	75	3 [7.35]	0.93	0.99	93	2 [2.38]	0.99	0.99
Tmax/Lmean	75	10 [10.95]	0.89	0.89	93	9 [8.98]	0.88	0.91
Tmax/Lmax	75	7 [9.31]	0.9	0.92	93	7 [7.11]	0.93	0.94

Values are presented as Spearman’s rank correlation coefficients (ρ) and Pearson’s correlation coefficients (r). All *p*-values were <0.0001. * Rank change (|ΔRank|) was defined as the absolute difference in lesion-wise rank between pre- and post-harmonization and is summarized as median [mean].

## Data Availability

The data presented in this study are not publicly available due to privacy and ethical restrictions related to patient data but are available from the corresponding author upon reasonable request.

## Abbreviations

The following abbreviations are used in this manuscript:

FDG	Fluorodeoxyglucose
PET	Positron emission tomography
Biograph 16	Biograph Sensation 16
Discovery IQ	Discovery IQ 4R
LSO	Lutetium oxyorthosilicate
BGO	Bismuth germanate
SiPM	Silicon photomultiplier
OSEM	Ordered subset expectation maximization
TOF	Time of flight
PSF	Point spread function
SUV	Standardized uptake value
MTV	Metabolic tumor volume
Tmax/Lmax	Tumor-to-liver maximum uptake ratio
Tmax/Lmean	Tumor maximum-to-liver mean uptake ratio.
EARL	European Association of Nuclear Medicine Research Ltd.
JSNM	Japanese Society of Nuclear Medicine
NEMA	National Electrical Manufacturers Association
RCs	Recovery coefficients
RMSE	Root mean square error
HNMM	Head and neck malignant melanoma
ACC	Adenoid cystic carcinoma
FWHM	Full width at half maximum
ROI	Region of interest

## Appendix A

### Appendix A.1. Methods: Phantom Acquisition Protocol

The NEMA NU2 image-quality body phantom used in this study contained six fillable hot spheres with inner diameters of 10, 13, 17, 22, 28, and 37 mm embedded in a quasi-cylindrical body cavity (280 × 210 × 180 mm). Phantom preparation procedures were identically performed for all scanners according to relevant JSNM guidelines [21]. Each hot sphere was filled with 18F-FDG at a sphere-to-background activity ratio of 4:1. All PET data were acquired in 3D list mode. For scanners with raw data access, 30-min acquisitions per bed position served as a low-noise reference, with additional routine acquisitions at 180 and 300 s. Biograph 16 phantom data (2018) were retrospectively analyzed, as the scanner is no longer in clinical use.

### Appendix A.2. Methods: Quantitative Analyses

#### Appendix A.2.1. SUV Measurements

SUVs were measured before and after harmonization to derive RCmax, RCmean, and noise metrics at each acquisition time (3, 5, and 30 min). Spherical volumes of interest (VOIs) for the hot spheres and background were manually placed by a nuclear medicine physician based on visual assessment using RAVAT software.

#### Appendix A.2.2. RC Evaluation

RCs were calculated as the ratio of measured SUV to the true activity concentration (target SUV ≈ 4.0 for the 4:1 sphere-to-background ratio). RCmax was defined as the maximum voxel value within the VOI. RCmean was calculated according to the EARL guidelines [31]:

$${RC}_{mean}=\frac{{SUV}_{mean}({VOI}_{T})}{{SUV}_{true}}$$

with the threshold T calculated as follow

$$T=0.5\times \left({SUV}_{max,\;sphere}-{SUV}_{mean,\;background}\right)+{SUV}_{mean,\;\;background}$$

Background SUVmean was obtained from twelve 37-mm spherical VOIs placed on the phantom’s central slice. RC values were assessed for compliance with the JSNM (3rd [JSNM3rd] and 4th [JSNM4th] editions) [14,21] and EARL (versions 1 and 2) standards [22].

#### Appendix A.2.3. RMSE Evaluation

The quantitative error was assessed using RMSE relative to an SUVtarget of 4.0. For each PET/CT system, acquisition duration, and FWHM, RMSE was calculated across sphere diameters (10–37 mm) as follows:

$$RMSE=\sqrt[{}]{\frac{1}{n}\sum_{i=1}^{n}{\left({SUV}_{i}-{SUV}_{target}\right)}^{2}}$$

where n is the number of spheres analyzed (diameters 10, 13, 17, 22, 28, and 37 mm). SUVi was defined as the SUVmean of each sphere measured using the VOI used for RC evaluation.

#### Appendix A.2.4. Evaluation of Image Quality and Noise

Image quality and background noise were evaluated before and after harmonization according to the JSNM procedure [21]. Accordingly, the obtained values were evaluated against the recommended criteria: percent background variability in 10-mm ROIs (N_10mm_) < 5.6% and background noise level background coefficient of variation (CV_background_) < 10%. In addition, the optional lesion detectability criterion, defined as percent contrast to background variability (Q_H,10mm_/N_10mm_) > 2.8, was assessed. For N_10mm_, twelve 10 mm ROIs were placed on the background of a central slice, with identical ROIs positioned 1 and 2 cm above and below, yielding 60 background ROIs. N_10mm_ was calculated as follows:

$$N_{10mm}=\frac{{SD}_{10mm}}{C_{B,10mm}}\times 100$$

SD_10mm_ represents the standard deviation of the mean activity concentration values measured across all 10 mm background ROIs. C_B,10mm_ is the average of the mean activity concentration values.

To evaluate Q_H,10mm_/N_10mm_, Q_H,10mm_ for a 10-mm hot sphere was calculated as follows:

$$Q_{H,10mm}=\frac{C_{H,10mm}/C_{B,10mm}-1}{a_{H}/a_{B}-1}\times 100$$

C_H,10mm_ and C_B,10mm_ denote the mean activity concentrations in the sphere and background, respectively. For the hot sphere, the three-dimensional VOI used for RC evaluation was applied instead of a two-dimensional ROI to maintain analytical consistency.

To evaluate CV_background_, circular 37-mm background ROIs were placed at the same slice positions as the 10-mm ROIs, yielding 60 ROIs. CV_background_ was calculated as follows:

$${CV}_{background}=mean\;of\left[\frac{{SD}_{37mm}}{C_{B,37mm}}\times 100\right]\left[\%\right]\left(N=60\right)$$

#### Appendix A.2.5. FWHM Adjustment for Harmonization

Post-reconstruction Gaussian filters were applied to harmonize RC values to the selected reference standard (JSNM3rd, JSNM4th, EARL1, or EARL2) [21,22,31]. Using RAVAT, FWHM was adjusted from 1 to 10 mm in 1-mm increments, and compliance was assessed at each setting. The smallest FWHM meeting the criteria across all acquisition durations was selected for each PET/CT system.

### Appendix A.3. Methods: Visual Assessment

Pre- and post-harmonization images from all PET systems and acquisition durations were visually assessed for lesion conspicuity by four readers: two nuclear medicine physicians (5 and 17 years of PET experience), one radiologist (13 years of radiology and 3 year of PET experience), and one first-year radiology resident without prior PET experience. Images were anonymized, randomly ordered by scanner, acquisition duration, and FWHM, and reviewed using a fixed SUV window (0–5) and standardized color map. Each hot sphere (10–37 mm) was graded on a 4-point scale (3, clearly visible; 2, visible; 1, barely visible; 0, not visible), and mean scores were calculated per condition. Readers received brief standardized instructions before evaluation. Inter-reader agreement was assessed using Fleiss’ κ (4-point scale) for all readers and for three excluding the trainee. The number of image datasets was predefined by the available phantom acquisitions; no formal a priori sample size calculation was performed.

## Appendix B

### Appendix B.1. Results: RC Evaluation

RCmax and RCmean of each PET/CT system were evaluated against the corresponding reference standards (JSNM and EARL) before and after harmonization [14,21,22]. Before harmonization, RC values substantially varied across PET/CT systems. No single reference standard encompassed the RC ranges of all four PET/CT systems. Biograph 16 showed the lowest RC values and complied only with the JSNM3rd criteria. Biograph Horizon satisfied the JSNM3rd and EARL1 criteria, whereas Discovery IQ met the JSNM4th criteria. The 10-mm RC values of Discovery MI exceeded all existing reference ranges. Representative examples of RCmax and RCmean for the 30-min acquisition are shown in Figure A1. Because Biograph 16 satisfied only the JSNM3rd RCmax criteria, we selected JSNM3rd as the common harmonization target. As the JSNM framework provides harmonization criteria for RCmax, but not RCmean, RCmax was used as the primary metric to determine harmonization filtering.

### Appendix B.2. Results: RMSE Evaluation

RMSE was evaluated using 3-min acquisitions as a clinical condition and 30-min acquisitions as a low-noise reference. Before harmonization (FWHM = 0 mm), Biograph 16 showed the highest RMSE, followed by Biograph Horizon, Discovery IQ, and Discovery MI. RMSE increased with greater FWHM. In 3-min acquisitions, values comparable to Biograph 16 were observed at 5–6 mm for Biograph Horizon and 7–8 mm for Discovery IQ; in 30-min acquisitions, at 6–7 mm and 7–8 mm, respectively (Figure A2). Discovery MI did not exceed Biograph 16 across the FWHM range (0–10 mm).

### Appendix B.3. Results: FWHM Adjustment for Harmonization

Table A1 summarizes the FWHM settings required to harmonize RC values across all sphere sizes to the JSNM 3rd edition reference range for each PET/CT system and acquisition duration. A single FWHM value that satisfied the JSNM 3rd edition criteria across all acquisition durations was identified as 4 mm for the Biograph Horizon, 9 mm for the Discovery IQ, and 8 mm for the Discovery MI. For the Biograph Horizon, RCmax at the 30-min acquisition was already within the JSNM 3rd edition reference range without additional smoothing; however, an FWHM of 4 mm was required to ensure that the 37-mm sphere acquired with the 3-min acquisition fell within the reference range. For the Discovery IQ, RC values for the 37-mm sphere acquired with a 3-min acquisition did not remain within the reference range until the FWHM was increased to 9 mm. As a result, a larger FWHM was required for the Discovery IQ compared with the Discovery MI.

**Table A1:** Explored and selected FWHM values for RCmax harmonization to the JSNM 3rd, across PET/CT systems.

System	Time (min)	Acceptable FWHM Range (mm)	Selected FWHM (mm)
Biograph Horizon	3	4~7	4
	5	0~7	
	30	0~5	
Discovery IQ	3	9	9
	5	5~9	
	30	6~9	
Discovery MI	3	8~10	8
	5	8~10	
	30	8~10	

### Appendix B.4. Results: Evaluation of Image Quality and Noise

Table A2 summarizes the image quality and noise metrics before and after harmonization across PET/CT systems. Regarding background variability, the recommended criteria for N_10mm_ and CV_background_ are <5.6% and <10%, respectively. Before harmonization, N_10mm_ exceeded this threshold in the 3-min acquisition of Biograph Horizon and in all acquisition durations (3, 5, and 30 min) of Discovery MI. After harmonization, N_10mm_ values for all PET systems and acquisition durations were within the recommended range. For CV_background_, all scanners at all acquisition durations satisfied the JSNM-recommended criteria before and after harmonization. For lesion detectability, Q_H,10mm_/N_10mm_ did not meet the recommended criterion (>2.8) for Biograph 16 at 3- and 5-min acquisition durations. This finding was considered to reflect the intrinsic detectability limitations of this scanner rather than the harmonization effect. For all other PET/CT systems, Q_H,10mm_/N_10mm_ exceeded 2.8 under all conditions before and after harmonization.

**Table A2:** Image quality and noise metrics before and after harmonization across PET/CT systems.

System	Acquisition (min)	FWHM for Harmonization	N_10mm_ (%)		CV_background_ (%)		Q_H,10mm_/N_10mm_	
			Before	After	Before	After	Before	After
Biograph 16	3	N/A	4.50	N/A	5.77	N/A	2.30	N/A
	5		3.39	N/A	7.58	N/A	1.60	N/A
	30		1.71	N/A	2.67	N/A	6.41	N/A
Biograph Horizon	3	4	6.01	4.93	8.58	7.35	3.17	3.46
	5		5.20	4.30	6.74	5.82	3.65	3.97
	30		2.14	1.78	2.88	2.49	10.54	11.32
Discovery IQ	3	9	5.51	1.80	8.37	3.84	4.73	5.67
	5		4.04	1.53	6.49	3.14	4.62	4.89
	30		1.79	0.86	3.21	2.06	11.58	9.67
Discovery MI	3	8	7.91	2.38	9.50	3.76	6.49	9.23
	5		6.17	1.79	7.20	3.03	8.39	12.23
	30		6.21	0.71	3.13	1.64	6.89	29.25

According to the JSNM guideline, the obtained values were evaluated against the recommended criteria for N_10mm_ < 5.6%, CVbackground < 10%, and Q_H,10mm_/N_10mm_ > 2.8 was assessed. Values not meeting the JSNM-referenced criteria are highlighted with a gray background.
